# Recent Advances on the Possible Neuroprotective Activities of Epstein-Barr Virus Oncogene BARF1 Protein in Chronic Inflammatory Disorders of Central Nervous System 

**DOI:** 10.2174/157015910792246191

**Published:** 2010-09

**Authors:** Alicia Wynne, Rupinder K Kanwar, Rajiv Khanna, Jagat R Kanwar

**Affiliations:** 1 Laboratory of Immunology and Molecular Biomedical Research (LIMBR), Centre for Biotechnology and Interdisciplinary Biosciences (BioDeakin), Institute for Technology & Research Innovation (ITRI), Deakin University, Geelong, Technology Precinct (GTP), Pigdons Road, Waurn Ponds, Geelong, Victoria 3217, Australia; 2 Australian Centre for Vaccine Development (NH&MRC), Queensland Institute of Medical Research, 300 Herston Road, Herston (Qld) 4006, Australia

**Keywords:** Epstein-Barr Virus, neuroregeneration, chronic inflammatory disorders, multiple sclerosis.

## Abstract

Multiple sclerosis and neurodegenerative diseases in which cells of the central nervous system (CNS) are lost or damaged are rapidly increasing in frequency, and there is neither effective treatment nor cure to impede or arrest their destructive course. The Epstein-Barr virus is a human gamma-herpesvirus that infects more than 90% of the human population worldwide and persisting for the lifetime of the host. It is associated with numerous epithelial cancers, principally undifferentiated nasopharyngeal carcinoma and gastric carcinoma. Individuals with a history of symptomatic primary EBV infection, called infectious mononucleosis, carry a moderately higher risk of developing multiple sclerosis (MS). It is not known how EBV infection potentially promotes autoimmunity and central nervous system (CNS) tissue damage in MS. Recently it has been found that EBV isolates from different geographic regions have highly conserved BARF1 epitopes. BARF1 protein has the neuroprotective and mitogenic activity, thus may be useful to combat and overcome neurodegenerative disease. BARF1 protein therapy can potentially be used to enhance the neuroprotective activities by combinational treatment with anti-inflammatory antagonists and neuroprotectors in neural disorders.

## INTRODUCTION

Neurodegenerative diseases, in which cells of the central nervous system (CNS) are lost or damaged, affect millions of people worldwide, and as population demographics change, the frequency of these age-associated diseases is increasing rapidly. In Australia, the percentage of individuals over the age of 65 is incessantly rising, at a rate greater than the growth of the population as a whole; a trend that is expected to persist for years to come [1]. Alzheimer’s disease, Parkinson’s disease, multiple sclerosis, trauma, epilepsy, stroke and many others fall under the title of neurodegenerative disease, and are linked by another common thread: there is neither effective treatment nor cure to impede or arrest the destructive course of these devastating illnesses. The shift in age distribution and the prospect of cases escalating to epidemic proportions highlights the necessity for intensive research in this field. There is a strong need for improved therapies and novel concepts for the control and management of neurodegenerative diseases. 

Extensive research has been carried out on the concept of neuroprotection, a strategy with the goal of reducing or preventing neuronal damage, apoptosis (programmed cell death), and the entry of inflammatory or autoreactive lymphocytes (B/T cells) through the blood brain barrier (BBB) [2]. Significant evidence suggesting the anti-apoptotic role of BARF1, a viral oncogene, indicates its potential as a neuroprotector [3, 4]. The added appeal of this viral gene is the prospective mitogenic properties it pertains, which may assist in overcoming the limitations of the slow growing nature of neurons [5]. 

Neurodegenerative diseases share several common mechanisms that lead to clinical symptoms, and for the purpose of this review, multiple sclerosis will be the focus, and a model for more detailed examination.

**Fig. (1):**
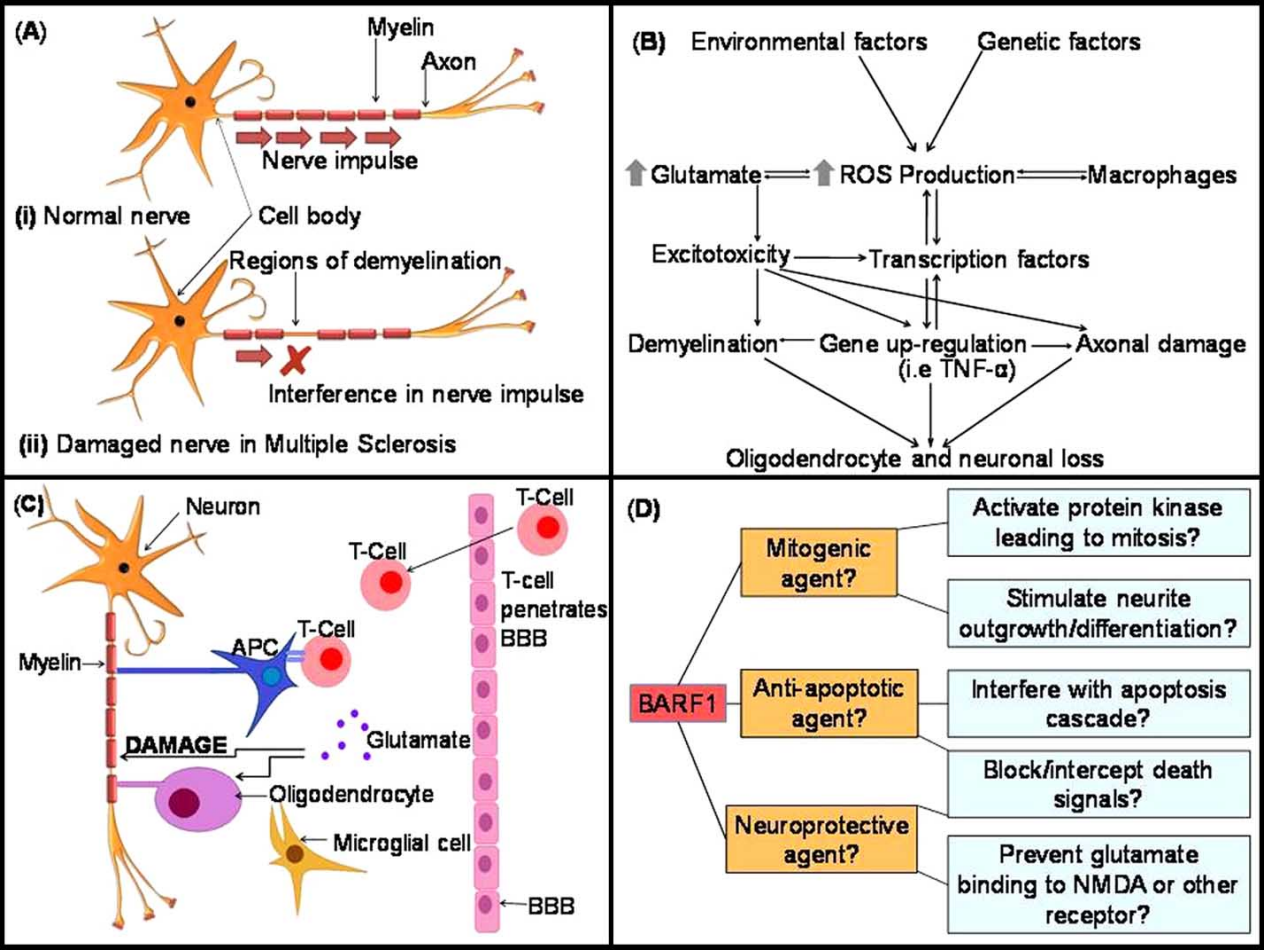
(**A**). Schematic of signal transduction in (**i**) a normal neuron in a healthy brain, versus (**ii**) a damaged demyelinated neuron in a patient affected by MS. In MS the immune system attacks the white matter of the brain in a combined insult involving auto-reactive T cells, B cells, macrophages and activated microglia. Inflammation leads to the formation of plaques, followed by the destruction of the protective myelin sheath. Myelin damage leads to impaired signal transduction or blockage, resulting in the clinical symptoms of MS. Removal or damage of the myelin sheath leaves the nerve axon exposed and subject to direct injury [24, 29, 31, 43]. (**B**). The primary sources of ROS and the cellular occurrences that may lead to oligodendrocyte and neuronal loss in EAE and MS. Modified from ref. [24]. (**C**). Autoimmune damage causes the supportive astrocytes to be lost or damaged, and repopulation by activated microglia follows. Activated microglia release glutamate, further increasing glutamate levels, which in turn promotes excitotoxic neuronal death by excessive NMDA receptor activation. The activation of T-cells specific against CNS-derived antigens follows. T-cells penetrate the BBB and release further glutamate [29, 39, 40, 45]. APC= Antigen presenting cell. (**D**). Possible effects of BARF1 protein on cells of the CNS and proposed mechanisms based on previous observations in a variety of cell types.

Multiple sclerosis (MS) and its animal model, experimental autoimmune encephalomyelitis (EAE) are chronic inflammatory demyelinating diseases affecting the CNS, in which the immune system attacks the white matter and eventually leads to disability and, at worst, paralysis [6]. While much research has been carried out on the progression of disease, the exact cause remains unclear. Histopathological studies have confirmed that MS lesions are the result of a combined insult, involving auto-reactive T cells, B cells, macrophages and activated microglia [6-8]. Inflammation in areas of the CNS results in the development of random ‘plaques’, and is followed by the destruction of the myelin sheath, a protective coating that surrounds and insulates the nerve axon. Myelin is vital for successful nerve signalling; its damage leads to impaired signal transduction or blockage, resulting in the clinical symptoms of MS (see Fig. **1A**) [2]. Once the protective sheath is removed or damaged, the nerve axon is left exposed and subject to direct injury. Axonal loss is the main determinant of permanent clinical disability, due to the limited ability of the CNS to regenerate [9]. 

MS can take the form of primary progressive (PPMS), relapsing progressive (RPMS), secondary progressive (SPMS) or relapsing remitting MS (RRMS), with the later being the most frequently occurring, responsible for 80% of cases. Both genetic and environmental factors are thought to increase the risk of disease [6]. The prevalence of MS ranges between 1 in 500 and 1 in 1500 of the population in Europe, North America, and Australasia [10]. No single effective drug has been developed to date, and current therapies are only partially effective. Two major causes of neurodegeneration and myelin degradation which may be targeted in MS therapy are glutamate insult and oxidative stress. 

### Oxidative Stress and Glutamate Damage in the MS Brain

“Reactive oxygen species” (ROS) are named due to their ability to cause oxidative changes within the cell [11]. Common cellular free radicals such as the hydroxyl radical (OH·), superoxide radical (O_2_ˉ·), and nitric oxide (NO·) are classified as ROS. Other reactive oxygen species such as hydrogen peroxide (H_2_O_2_) and peroxynitrate (ONOO) also come under the title, because although not free radicals themselves, they can lead to the generation of such through common chemical reactions [12]. ROS are by-products of aerobic metabolism, and are also generated as second messengers in certain signal transduction pathways. Cells may also produce ROS in response to an environmental insult, or take up ROS directly from the extracellular fluid. In fact, ROS can be created as by-products of many electron transfer reactions within the cell or its surrounds, however the major source is the mitochondria, as its consumption constitutes around 85% of O_2_ used by the cell. The generation of ROS in mitochondria is due to inhibition of the electron transport chain, and once generation begins, a positive feedback mechanism may occur where existing ROS further hinder electron transport, amplifying ROS production. Plasma membrane oxidases can also generate ROS during physiological signaling [6, 11]. 

Production of ROS is an essential component of some signal transduction pathways, and is crucial for normal cell function. ROS are also thought to be involved in the direct regulation of transcription factor activity, one example being oxidation-reduction of cysteine residues in the DNA binding domain [13]. However, during unregulated ROS production, excessive or fixed levels of ROS can result in acute damage to cellular components, and may lead to cessation of mitochondrial energy production. Several antioxidant enzymes defend the cell against oxidative damage, the most important being catalase, glutathione peroxidase, Cu, Zn-superoxide dismutase (CuZuSOD) and Mn-superoxide dismutase (MnSOD). These are the body’s natural mechanism of protection from oxidation; however these are not always sufficient to prevent cellular injury, and may become damaged. When the ROS production outweighs these enzyme’s ability to defend, the result is known as ‘oxidative stress’ (OS) [6, 11]. 

Several characteristics of the central nervous system increase its susceptibility to oxidative stress. The CNS exhibits a high metabolic rate and increased ATP synthesis, as well as high lipid content and the utilization of dopamine oxidation and reactions involving glutamate. The CNS also utilizes a large amount of molecular oxygen, and has a limited ability for cellular regeneration. These characteristics equate to a heightened risk of damage by ROS, and such damage has been shown to be implicated in several neurodegenerative diseases, as well as neural deterioration in the natural ageing process [11]. OS may result in subsequent cell death through apoptosis (programmed cell death) and neurodegeneration in the CNS due to oxidation of proteins, lipids and DNA [12]. Fig. (**1B**) [6] shows the relationship between oxidative stress, glutamate and the CNS. 

There are various factors contributing to OS in cells, however the neurotransmitter glutamate is the primary effector within the brain, mainly through the activation of ionotrophic receptors. The role of glutamate is illustrated in Fig. (**1C**) [14]. Glutamate and related amino acids are released by approximately 40% of all synapses in the nervous system, and are responsible for the majority of excitatory synaptic activity in mammals [15]. Ionotrophic receptors include the main glutamate receptor N-methyl-D-aspartate (NMDA), α-amino-3-hydroxy-5-methyl-4-isoxasoleproprionic acid (AMPA) and the kainic acid (KA) family of receptors. In MS, glutamate-related excitotoxicity, caused by excessive activation of these receptors (leading to a Ca^2+^ overload), is responsible for neuronal and oligodendrocyte death [2, 16, 17]. In addition, microglia, the resident macrophages of the CNS, become activated by increased glutamate concentration. Activated microglia proliferate, secrete cytokines, chemokines, nitric oxide and ROS, and may become phagocytic; outcomes all of which cause further injury to the ailing CNS [9]. Oligodendrocytes have been found to be particularly susceptible to glutamate excitotoxicity, *via *the AMPA/kainate receptors. AMPA/kainate antagonists have been shown to increase oligodendrocyte survival as well as reducing axonal damage [16, 17]. These findings have led to the introduction of treatments that block glutamate neurotransmission, namely riluzole, which is now a treatment for autoimmune demyelination [6, 18]. 

### Current Therapies 

Several disease-modifying therapies (DMTs) are currently available for the treatment of MS, including two formulations of interferon (IFN)-β-1a (Avonex^®^ and Rebif^®^), interferon (IFN)-β-1b (Betaseron^®^), glatiramer acetate (GA) (Copaxone^®^), and natalizumab (Tysabri^®^). Due to the clinical variability and unpredictable nature of MS, prescribing treatments for specific individuals is complicated, and while all of the above were shown to be partially effective during clinical trials, no therapy has the capability to halt disease progression [19-21]. Novel neuroprotective and restorative strategies are undergoing animal studies and clinical testing, such as chemokine-receptor antagonists, which reduce the entry of lymphocytes to the CNS, and blockers of neurite outgrowth inhibitor, which promote axonal sprouting [22]. 

An amalgamation of cytotoxic cytokines, autoantibodies, toxic levels of glutamate and ROS cause damage to myelin, neural axons and oligodendrocytes, resulting in the clinical symptoms of MS [7]. To successfully halt progression or reverse MS, a treatment must simultaneously target a combination of these factors. The α4 and β7 integrins are thought to be significant contributors in the development of MS, playing possible roles in inflammation and migration of leukocytes subpopulations across the BBB, and treatment with anti- β7 and α4 integrin subunit antibodies has led to accelerated improvement and more complete remission in EAE subjects [23, 24]. Kanwar *et al.* [2, 7] devised an approach which combines neuroprotection with blockage of inflammation using MAdCAM-1 antibody (an antibody to the ligand for α4β7), neuroprotector glycine-proline-glutamic acid (GPE) and the (AMPA)/kainate receptor antagonist 2, 3-dihydroxyl-6-nitro-7-sulfamoylbenzo(f)quinoxaline (NQBX). The method has shown promise in the treatment of both early and advanced stage unremitting EAE, resulting in amelioration of disease and repair of the CNS, gauged by increased oligodendrocyte survival and remyelination, as well as reduced inflammation, apoptosis and axonal damage [7]. Administration of either a combination of NQBX and GPE or preferably all 3 reagents (NQBX, GPE and anti-MAdCAM-1) reduce the expression of nitric oxide as well as several proinflammatory and immunoregulatory cytokines, in particular IL-6, which plays an important role in mediating EAE. Subjects showed discernible improvements in every physical feature examined for 5 weeks, but relapsed after suspension of treatment suggesting a requirement for ongoing treatment [7]. Further exploration and experimentation using a multi-faceted approach, inhibiting inflammation while simultaneously protecting neurons and oligodendrocytes, may lead to the development of effective therapies for the treatment of MS. 

### Epstein-Barr Virus and the BARF1 Oncogene

The Epstein-Barr virus (EBV) belongs to family of herpes viruses, which persistently infects over 90% of the global population [5, 12]. The initial infection usually goes unnoticed if contracted in early childhood, but may induce infectious mononucleosis if the first contact occurs during adolescence or adulthood. The virus remains in B cells for the life of the individual, and has shown the ability to immortalise the lymphocytes *in vitro* [5]. EBV has been linked to both nasopharyngeal carcinoma (NPC) and Burkitt’s lymphoma (BL) and is also thought to be associated with subsets of other types of carcinomas, such as gastric carcinoma and lymphoepithelioma-like carcinoma in the salivary glands and the thymus [25]. EBV has been detected in certain types of breast tumours, and although it is unlikely to play a primary etiologic role in breast cancer, it may contribute to tumour progression [26-28]. The virus has also been shown to confer resistance to the chemotherapeutic drug Taxol and to induce over-expression of the multidrug resistance gene MDR1. It is thought that EBV may cause changes in the phenotype of a subpopulation of tumour cells, resulting in more aggressive behavior [3, 28]. While many links between EBV and cancer have been established, the pathogenic role of the virus remains poorly understood. 

EBV is also hypothesised to trigger several autoimmune diseases [29, 30]. Credible prospective seroepidemiological studies have consistently verified the relationship between EBV and multiple sclerosis, with close to 100% of MS patients being seropositive for the virus [20, 29, 31, 32]. EBV has been speculated as a critical factor for the development and progression of MS [20, 29], but its role in the etiology and pathogenesis is not well defined. Consistent with suspicion, analyses have shown that the risk of developing MS significantly worsens with increased EBV antibody titer, in particular, immunoglobulin G (IgG) antibodies binding to EBV nuclear antigen 1 (EBNA1) [29, 33]. While several possible scenarios regarding the relationship between MS and EBV have been proposed, nothing has been substantiated and understanding is lacking [29]. 

The universal positivity of MS patients for EBV is well established; hence it is feasible, in terms of treatment, to exploit an element of the virus itself. A search for a component or product of EBV that could be used to the patient’s advantage identified a stand-out: BARF1 protein. Several attractive properties of BARF1 allude to its potential for therapeutic use.

Approximately 90 genes constitute the EBV genome, some of which have been studied in depth, with research showing strong evidence for oncogenicity [3, 25, 34, 35]. Oncogene functions are generally considered to be cell-type specific, exhibiting varied effects on different cell lines. While the four EBV nuclear antigens EBNA 1, EBNA 2, EBNA 3A and EBNA 3C and the latent membrane protein LMP1 are required for the immortalisation (continuous growth and division) of B lymphocytes *in vitro*, it is the BARF1 gene that is thought to confer this property in epithelial kidney cells and possibly gastric cancer cells [3, 34, 36, 37]. In rodent cells and human B cell lines Loukes and Akata, however, BARF1 appears to induce malignant transformation, the conversion of a previously immortalized cell into the malignant phenotype [3]. LMP1 and BARF1 both appear to be important effectors in NPC, with the genes being expressed in 50% and 90% of cases respectively [5]. It is thought that the expression of BARF1 may allow generation of further ectopic growth or progression to a more advanced stage in gastric cancers, and likely plays a similar role in other tumours [3]. 

BARF1 is an early gene, transcribed shortly after EBV infection from the BAMH1 A fragment of the genome [38]. The product is a secretory glycoprotein, consisting of two immunoglobulin-like domains [5]. Tarbouriech *et al.* [5] characterised the structure of BARF1, and found that it was most closely related to human CD80 (also known as B7-1), and has the same topology as the co-stimulatory molecule. BARF1 is thought to have been derived from CD80 during evolution. Expression cloning has found that BARF1 is a functional homologue of the human colony-stimulating factor receptor, *c-fms*, allowing it to bind to the hCSF-1 ligand. Binding reduces the action of the cytokine on the proliferation of macrophages [38], as well as the interferon production by mononuclear cells [39]. This indicates that BARF1 protein may also play an immunomodulating role *in-situ* [5]. BARF1 protein has induced cell cycle activation of Balb/c3T3 fibroblasts, human B-cells and primary monkey epithelial cells, suggesting it may have mitogenic activity on certain cells types [5]. Other research has indicated that BARF1 may also act as a survival factor, by either suppressing apoptosis or promoting cell proliferation in cell lines [3]. Wang and Tsao *et al.* [3] found that BARF1 expression promotes survival and may confer protection *via *the inactivation of apoptosis pathways. Reduced apoptosis was associated with the increased Bcl-2 to Bax ratio, a regulator of cell death, which acts by activating or inhibiting procaspase activity.

### Neurogenesis and Neuroregeneration: The Possibilities and Constraints

One limitation in the treatment of neurodegenerative diseases such as MS is the ability of the CNS to regenerate damaged or lost neurons. While axonal regeneration occurs readily in the peripheral nervous system, it does not take place in the CNS to any great extent. This is due to a number of factors, but the most prominent is a non-permissive growth environment and a lack of appropriate growth factors [40]. Spanish scientist and 1906 Nobel Prize winner Ramon y Cajal, famous for his pioneering work on the CNS, quoted ‘in adult centres, the nerve paths are something fixed, ended and immutable. Everything may die, nothing may be regenerated’. This long-standing dogma has been continuously challenged, and it is now known that neural progenitors and stem cells in the adult CNS can generate new neurons, astrocytes and oligodendrocytes in the subventricular zone (SVZ)/olfactory bulb and the dentate gyrus subregion of the hippocampus [41]. While this discovery is now widely accepted as fact, some controversial claims have been made relating to the induction of division in mature neurons. Brewer [42] declares that mature neurons were effectively regenerated in the cerebral cortex of adult rats, while Gu *et al.* [43] proliferated adult CNS neurons in a specialised culture [42, 43]. However, the general consensus is that neuron morphology and neurochemical complexity is unreasonably intricate to be maintained during DNA replication and nucleus remodeling, although division *is* theoretically possible [41]. Nonetheless, while most reports suggest that induction of a mature neuron to re-enter the cell cycle ends in failure, the possibly is intriguing, and successful division of a differentiated neuron would be a breakthrough in neuroscience. 

Despite the slim possibility of differentiated neuronal division *in vivo*, the hope of successful generation, insertion and functional integration of new neurons lies with neural progenitors and precursors. The manipulation of these cells could give rise to exciting new therapies for the treatment of degenerative diseases of the brain. Neuronal replacement is imperative for full reversal of advanced neurodegenerative disease, and the ultimate goal in disease therapy is to wield neuronal precursors towards neuronal cell repopulation. Some major complications, however, are the limited number of resident progenitor, precursor and stem cells in the neuron-producing regions of the brain, their moderate divisional activity, and the slow growth of progeny. It would thus be feasible to employ a growth factor or mitotic agent, to encourage the production and enhance the yield of newborn neurons. 

### The Therapeutic Potential of BARF1 Protein

The previously identified properties of BARF1 protein entertain the following question: Does this protein have potential for therapeutic use as a survival factor/mitogen/neuroprotector to treat multiple sclerosis or other neurodegenerative or autoimmune diseases? Studies describing the neuroprotective ability of BARF1 or use of the protein in the CNS are yet to be published, however as discussed previously, past studies have found other neuroprotectors such as GPE and NQBX useful in treating EAE in its early stages [7]. While BARF1 protein may act as a survival factor on some cell types such as gastric cancer cells, it is not known if it will exhibit the same properties when applied to neurons and other cells of the nervous system. If it does play and anti-apoptotic role in the CNS, this property, combined with its possible mitogenic ability, could prove invaluable for the treatment of MS. By up-regulating of the production of BARF1 protein in MS patients, it may be possible to protect components of the CNS against damage inflicted by the immune system, cytokines, ROS and glutamate. The possible effects of BARF1 protein on cells of the CNS and proposed mechanisms are outlined in Fig. (**1D**). Hence, the recognised properties of BARF1 protein suggest that it may be ideal for such application. If BARF1 behaves it the way we anticipate, acting as a mitotic and anti-apoptotic agent, it may simultaneously protect neurons from stimuli-induced apoptosis, while encouraging the growth and proliferation of neurons and glia. By incorporating BARF1 into a multi-faceted treatment such as that devised by Kanwar *et al*. (2004), and either upregulating its production or using nanoparticle delivery, it may prove to be a favourable option indeed, incorporating two desired properties into a single therapeutic component. Immortalization, malignant transformation and immunomodulation are possible properties of BARF1 which may prove troublesome in upregulation or vector delivery. Due to the inflammatory origins of MS, all care must be taken so as not to intensify MS pathology, as blocking the proliferation of macrophages and interferon may contribute to such. The oncogenic nature of BARF1 also implies that it may contribute to tumour cell formation, and while the prevention of apoptosis in cells of the CNS may be beneficial in neurodegerative disease, it is also possible that BARF1 will prevent cancerous cells from undergoing programmed death, but rather allow them to survive and proliferate. Despite these risks, the properties of BARF1 appear particularly appealing for applications in neurodegenerative disease and while we must take due caution, the likely benefits suggest promising results. 

### A Model for CNS Repair

Marsupials are born considerably immature and much of their development occurs postnataly. Therefore, species such as the North American opossum *Didelphis virginiana* and the short-tailed Brazilian opossum *Monodelphis domestica* serve as attractive models in which to study the development and repair of motor systems [44-46]. Because the opossum in a foetal-like state only 12 days after conception, it possible to transect or crush the spinal cord early in development without sacrificing the pregnant opossum or performing intrauterine surgery [44]. This allows observation of CNS repair mechanisms, furthering knowledge in this area which could possibly lead to improved treatments of spinal trauma patients and CNS damage. 

Mammalian experiments involving transection of the spinal cord of opossum *Didelphis virginiana *pups have resulted in normal functionality and use of hind limbs at maturity [46]. When the thoracic spinal cord was transected at postnatal day (PD) 5, axons forming all major descending and ascending tracts bridge the lesion site. Such growth resulted in remarkably normal hind limb movement in adulthood. Similar experiments involving opossum pups at PD 20 showed growth of descending axons through the transected site, but no growth of ascending axons. The pups transected at PD20 also showed abnormal functionality and uncoordinated hind limb movement and loss of sensation caudal to the lesion. Other experiments involving crushing of the thoracic spinal cord on PD7 in *Monodelphis* resulted in normal spinal cord development and functionality at 3 months and at maturity. As cell division occurs naturally within the developing spinal cord (without transection or crushing), neurogenesis and gliogenesis observed in these experiments are not solely in response to injury. However, increased proliferation in response to lesion was apparent in the meninges, but it is not known if meningeal cells contribute to cord reconstruction [46]. Spinal cord tissue regeneration in lower vertebrates has been well documented and is dependent on the ependymal cell proliferation, as these cells bridge the gap at the site of damage before differentiating into new neurons and glia [47-51]. There have been reports of mitosis in cells which line the central canal after spinal cord injury in postnatal and adult mammals, but there is little reconstitution of new tissue [52, 53]. 

The proliferation of multipotent stem cells in the mammalian CNS is dependent on certain growth factors [54]. The application of such growth factors, as well as possible mitogenic agents such as BARF1, to the spinal cord lesion of these experimental models may result in enhanced reconstruction and possible improved functionality. By understanding the specialised growth environment in which the nervous system develops and recreating such in a damaged CNS, it may be possible to induce repair in Multiple sclerosis patients and those with other neurodegenerative disorders, as well as spinal trauma patients.

### Alternative Therapies and Future Direction for CNS Repair

Stem cell transplantation, of either embryonic or adult origin, represents a promising alternative for the treatment of MS [55]. While there are some issues regarding the therapeutic use of embryonic stem cells, such as feeder-independent growth (expansion) and in-vivo teratocarcinoma formation, this avenue of treatment is under investigation and experts remain positive. Adult or somatic stem cells have been widely used in experimental and clinical settings showing no significant toxicity or side effects, and without tumour formation. Somatic stem cells also appeal due to their ready-to-use nature, being derived from different tissues such as the brain [55]. 

A major constraint for stem cell transplantation regards the route of administration, which very much depends on the lesion site, be it focal or multifocal. Focal CNS disorders such as Parkinson’s disease, acute spinal cord injury, brain trauma and stroke exhibit anatomo-pathological features and may respond to intralesional cell transplantation. The multifocality of disorders such as MS and epilepsy require a different approach. Systemic transplantation such as intravenous and intrathecal approaches allow delivery of stem cells *via *the blood stream or cerebrospinal fluid circulation. In these methods the transplanted cells follow a gradient of chemo-attractants such as chemokines and pro-inflammatory cytokines which occur at the site of inflammatory lesions [55-57]. Successful delivery of transplanted neural precursor cells (NPCs), hematopoietic (HSCs) and mesenchymal stem cells (MSCs) has been achieved in EAE models of MS [55, 56, 58], as well as spinal trauma [59, 60], epilepsy and stroke [61, 62]. The capacity of stem cells to migrate to inflamed areas of the CNS and tether, roll and adhere to the inflamed endothelial cells is credited to the expression of cell adhesion molecules (CAM) and chemokine receptors [55, 56]. 

Transplantation of myelin-forming cells have remarkable remyelinating properties, resulting in the restoration of electrophysical nerve function [63], however, the mechanisms as to how these transplanted cells exert a beneficial clinical effect in EAE are unclear. Mature astrocytes have exhibited anti-inflammatory properties *in vitro*, but did not migrate to the brain following intraventricular transplantation, and therefore did not reduce brain inflammation or affect the clinical course of EAE [64, 65]. The clinical and pathological effects of transplanted cells are dependent on their ability to migrate to the active inflammatory demyelinated regions. Effective migration of NPCs enable their delivery to and contact with inflamed tissue, causing suppression of inflammation and reduction of demyelination and axonal injury [66]. Transplantation of neurospheres has been shown to attenuate the brain inflammatory process in EAE and reduce demyelination and axonal injury [66].

Replacement therapies alone may be a satisfactory treatment for accident patients suffering from spinal trauma and those who have experienced one-off damage such as stroke sufferers; however sufferers of chronic and progressive diseases (MS, Parkinson’s disease etc.) require a more complex approach. These patients would need ongoing replacement therapy to repair the continual damage afflicted by disease, for example oxidative stress and glutamate damage in MS. It seems the key to successful CNS repair requires a multi-faceted approach, combining neuroprotection and blockage of inflammation with stem cell replacement or proliferation therapies. The benefits of stem cell transplantation may be enhanced by combining this therapy with a neuroprotective, anti-apoptotic and/or a mitogenic agent. BARF1 protein, perhaps possessing two of the above properties of both neuroprotection and mitogenic activity, seems an obvious candidate in such an approach. With the focus on not one but a variety of avenues of treatment, it may be possible to combat and overcome neurodegenerative disease. Another benefit of the described approach relates to the heterogeneous nature of MS: it differs considerably between patients and disease categories [2]. The individual components of treatment may be varied and tailored for an individual or group; a huge benefit in such a diverse and destructive disease. While considerable research has been carried out on a diversity of cell-based therapies and some neuroprotective and anti-inflammatory treatments, thorough in-vivo studies must completed before all-purpose treatments can become a reality.

## AUTHOR DISCLOSURES

Alicia Wynne, Rupinder K. Kanwar, Rajiv Khanna and Jagat R. Kanwar, have no conflicts of interest.
